# When Silicone Oil Goes Anterior: A Case of Anterior Segment Neovascularization After Complex Retinal Detachment Repair

**DOI:** 10.7759/cureus.115798

**Published:** 2026-09-04

**Authors:** Emily Rodman, Mariana Ferraz, Julie A Rodman

**Affiliations:** 1 Osteopathic Medicine, Nova Southeastern University Dr. Kiran C. Patel College of Osteopathic Medicine, Fort Lauderdale, USA; 2 College of Optometry, Nova Southeastern University, Fort Lauderdale, USA

**Keywords:** emulsification, intralenticular neovascularization, neovascularization of the iris, retinal detachment, silicone oil

## Abstract

Silicone oil is widely used for intraocular tamponade in complicated retinal detachment surgery. Despite the efficacy of this treatment, complications may occur, including emulsification of the silicone oil in the anterior chamber. Chronic ischemia from recurrent retinal detachment or the development of proliferative vitreoretinopathy places an eye at risk for the development of iris rubeosis and/or intralenticular neovascularization.

An 81-year-old pseudophakic female presented with a chief complaint of blurry vision in the left eye (OS) with no light perception in the right eye (OD) since retinal reattachment surgery OD. Review of her ocular history, including outside ophthalmic records, documented pars plana vitrectomy with silicone oil tamponade for retinal detachment complicated by grade C proliferative vitreoretinopathy. She was pseudophakic in both eyes, with a history of complicated cataract extraction and a compromised posterior capsule in the right eye. The silicone oil migrated into the anterior chamber via a compromised posterior capsule. She subsequently developed iris and intralenticular neovascularization. Silicone oil tamponade with vitrectomy is a standard procedure used for complicated retinal detachments associated with proliferative vitreoretinopathy, proliferative diabetic retinopathy, complex retinal tears, or ocular trauma. However, complications have commonly been reported. We present a patient who exhibited silicone oil emulsification with concomitant neovascularization of the iris and lens secondary to ischemia from complicated retinal detachment repair.

## Introduction

Due to its unique viscoelastic properties, silicone oil has been successfully used as a vitreous substitute in the management of tractional retinal detachments, giant retinal tears, traumatic retinal detachments, and complex retinal detachments due to proliferative vitreoretinopathy. The tamponade effect is produced by interfacial surface tension at the oil-aqueous interface, which helps seal the retinal break and prevent fluid from passing through it, while buoyancy helps maintain the tamponade against the retina [1]. For inferior retinal breaks, it is important to note that conventional silicone oil provides primarily superior tamponade. Heavy silicone oils have been developed to provide more effective tamponade for inferior retinal pathology [2].

Anterior segment neovascularization, particularly iris rubeosis, may develop in eyes with chronic posterior segment ischemia associated with retinal detachment and proliferative vitreoretinopathy. Ischemic retinal tissue can release angiogenic mediators, including vascular endothelial growth factor (VEGF), which can diffuse anteriorly and promote neovascularization of the iris and other anterior segment structures [3]. Intralenticular neovascularization is considerably less common because the normal lens is avascular; however, disruption of the lens capsule from trauma or surgery may facilitate vascular ingrowth into the lens or capsular region [4-6].

We report a rare case of concomitant iris and intralenticular neovascularization in an eye with chronic retinal detachment and proliferative vitreoretinopathy following silicone oil tamponade, with associated anterior migration and emulsification of silicone oil.

## Case presentation

An 81-year-old woman presented with decreased vision in the left eye (OS) and longstanding profound vision loss in the right eye (OD). The duration of her visual symptoms was unclear. She reported that the severe vision loss in OD began following retinal detachment repair. Review of her ocular history, including outside ophthalmic records, was significant for bilateral cataract extraction, retinal detachment repair with pars plana vitrectomy and silicone oil tamponade OD, grade C proliferative vitreoretinopathy OD with full-thickness retinal folds, scleral buckle placement OD, and exudative age-related macular degeneration OS with macular scarring (Figure 1). The left eye was being treated with intravitreal anti-VEGF injections (bevacizumab [Avastin]) at four-week intervals. She was also using Cosopt (dorzolamide/timolol) bid OD, as she had reported developing “high pressures” in that eye after the surgery. Her medical history was significant for hypertension and hypercholesterolemia. She denied diabetes, carotid artery disease, or ocular ischemic syndrome. Her medications included apixaban (Eliquis) 5 mg twice daily, sacubitril/valsartan (Entresto) 24/26 mg twice daily, and metoprolol tartrate 100 mg daily. The indications for these medications were not available in the records reviewed.

**Figure 1:**
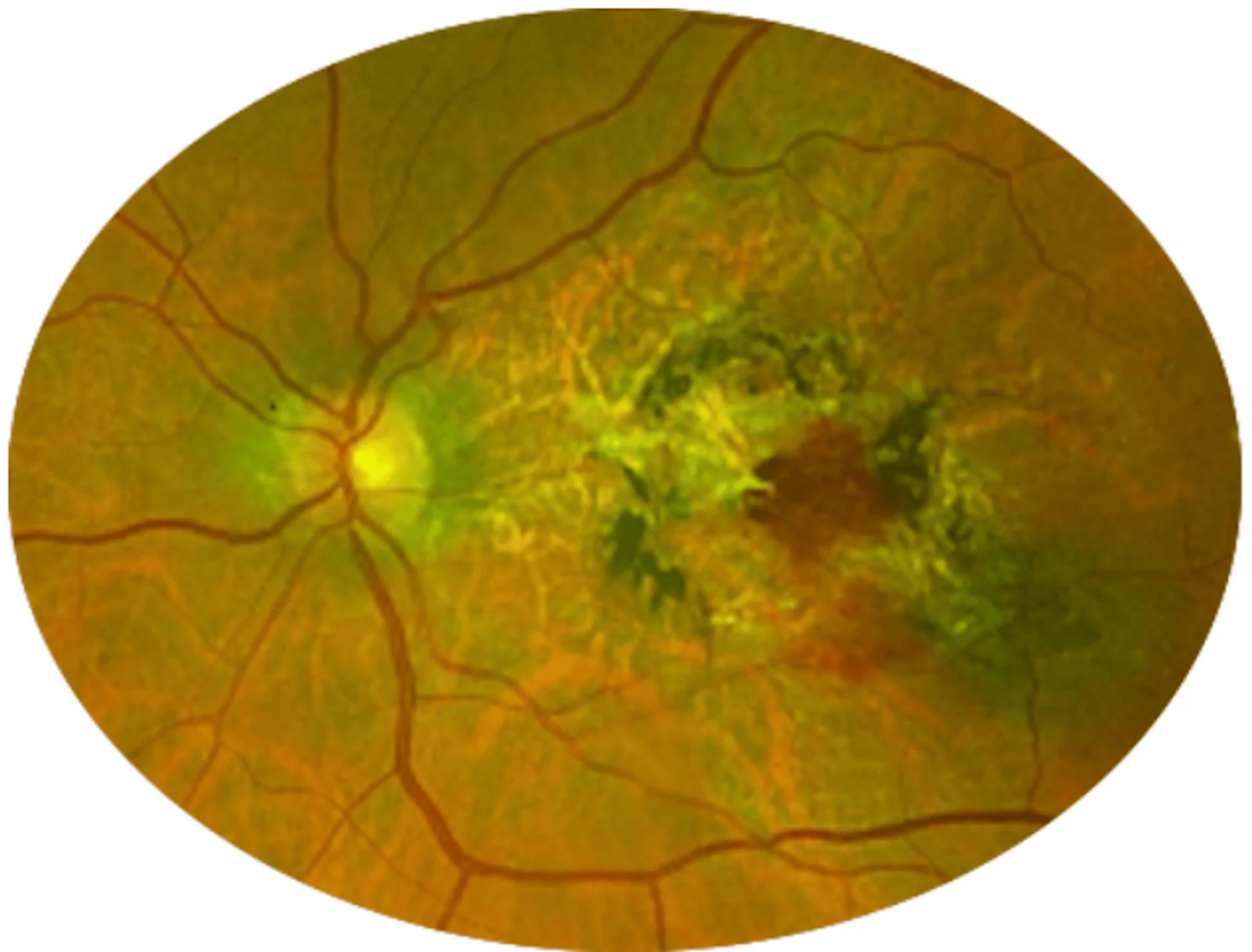
Fundus photograph of the left illustrating active macular neovascular membrane with surrounding chorioretinal scarring.

Visual acuity was no light perception (NLP) OD; best corrected visual acuity was 20/125 OS. The patient had a small hyperopic prescription OS and wore reading glasses. The right pupil was amaurotic and non-reactive to light, consistent with the patient's NLP status. Pupil morphology was round, with no evidence of ectropion uveae or posterior synechiae. Extraocular motility was full in both eyes. Confrontation visual fields could not be assessed in OD because of no light perception. Slit-lamp biomicroscopy revealed unremarkable lids and lashes as well as a clear cornea. The anterior chamber was quiet without cells or flare OS. Iris rubeosis and intralenticular neovascularization were noted OD, with silicone oil emulsification in the anterior chamber obscuring the view of the intraocular lens (Figures 2-3). There was a clear view of the posterior chamber intraocular lens OS without evidence of rubeosis. Intraocular pressure was measured at 3 mmHg OD and 10 mmHg OS by Goldmann applanation tonometry. The right-eye measurement was repeated and remained 3 mmHg. Although the patient reported chronic use of Cosopt (dorzolamide/timolol) OD for previously elevated intraocular pressure, the degree of hypotony was considered disproportionate to topical aqueous suppression alone. Given the history of chronic retinal detachment and grade C proliferative vitreoretinopathy, the marked hypotony raised concern for underlying ciliary body dysfunction or shutdown, anterior proliferative vitreoretinopathy with cyclitic membrane formation, or chronic ciliochoroidal detachment. The patient reported experiencing periocular pain when she had previously attempted to discontinue Cosopt.

**Figure 2:**
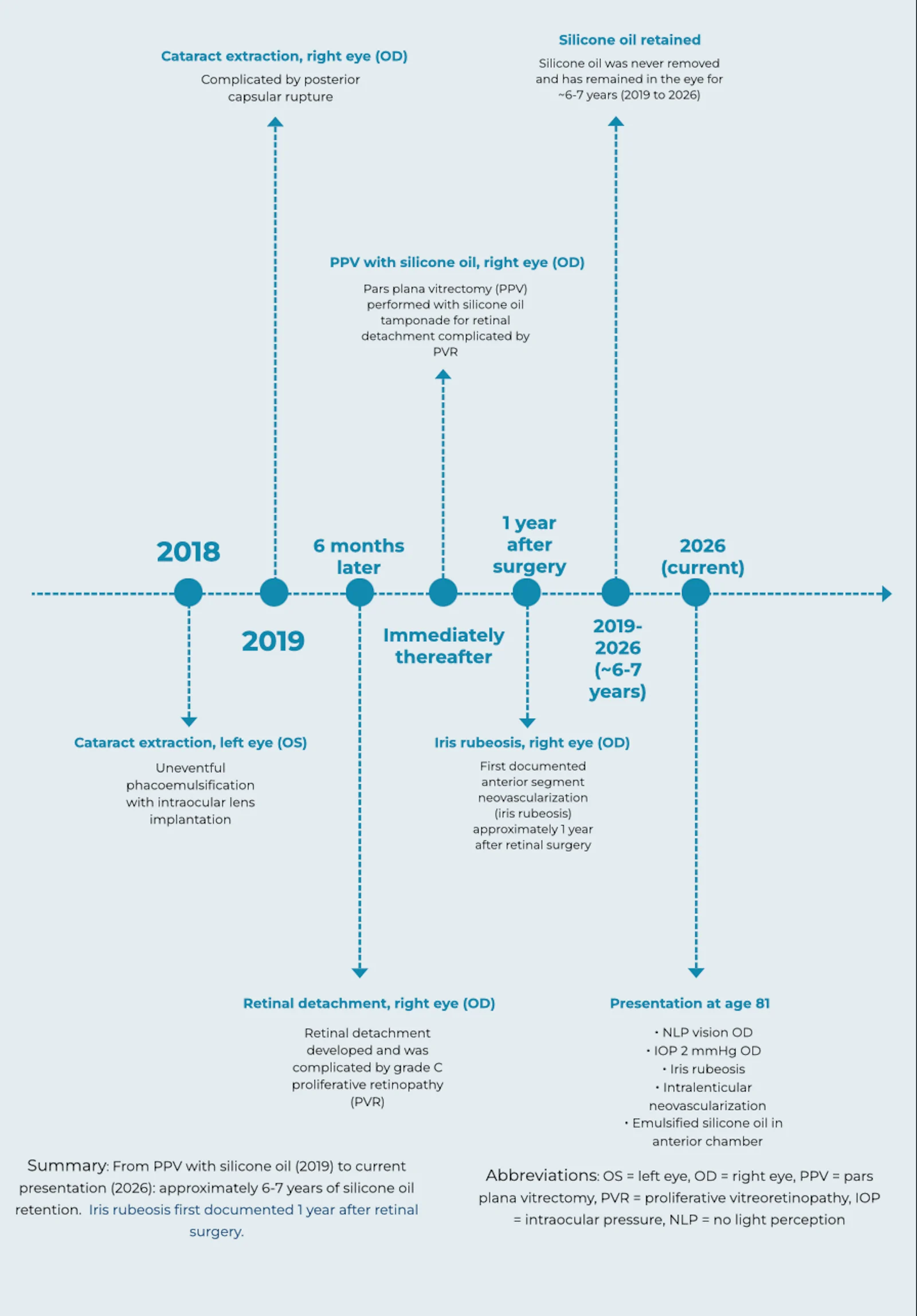
Chronological timeline of patient's ocular surgical history.

**Figure 3:**
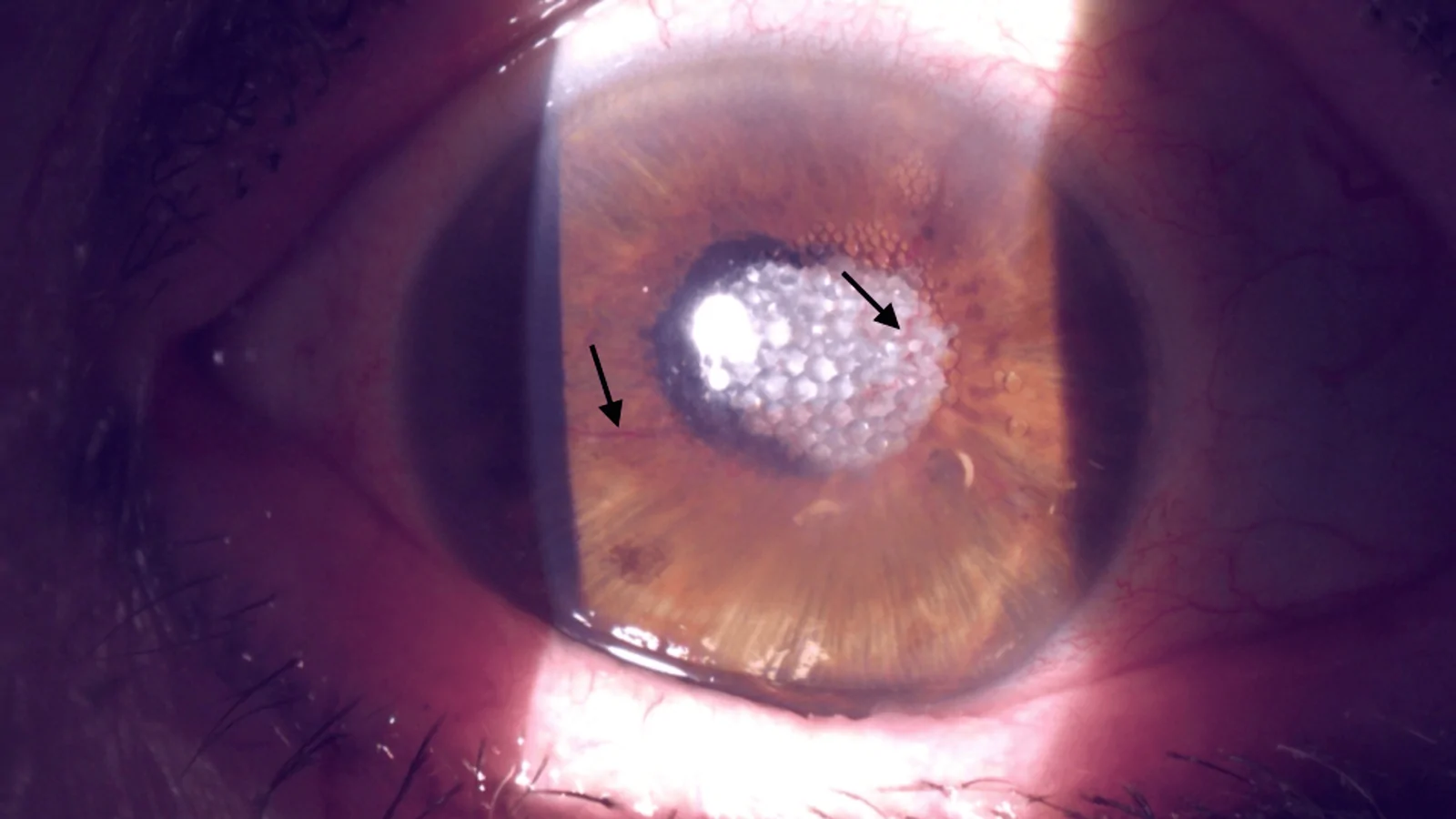
Slit-lamp photograph of silicone oil emulsification with iris rubeosis and intralenticular neovascularization (10x magnification).

The optic nerve was pink and distinct, with a cup-to-disc ratio of .40H/V in the left eye with evidence of macular neovascularization and diffuse chorioretinal scarring (Figure 4). There was no view of the retina OD. To further evaluate the posterior segment and assess for possible choroidal or ciliary body abnormalities, B-scan ultrasonography was recommended; however, the patient declined further imaging, stating that she had recently been evaluated by her retinal specialist and did not want to repeat testing. Anterior segment optical coherence tomography was also considered to further characterize the anterior segment and the location of the neovascularization; however, these studies were not performed.

**Figure 4:**
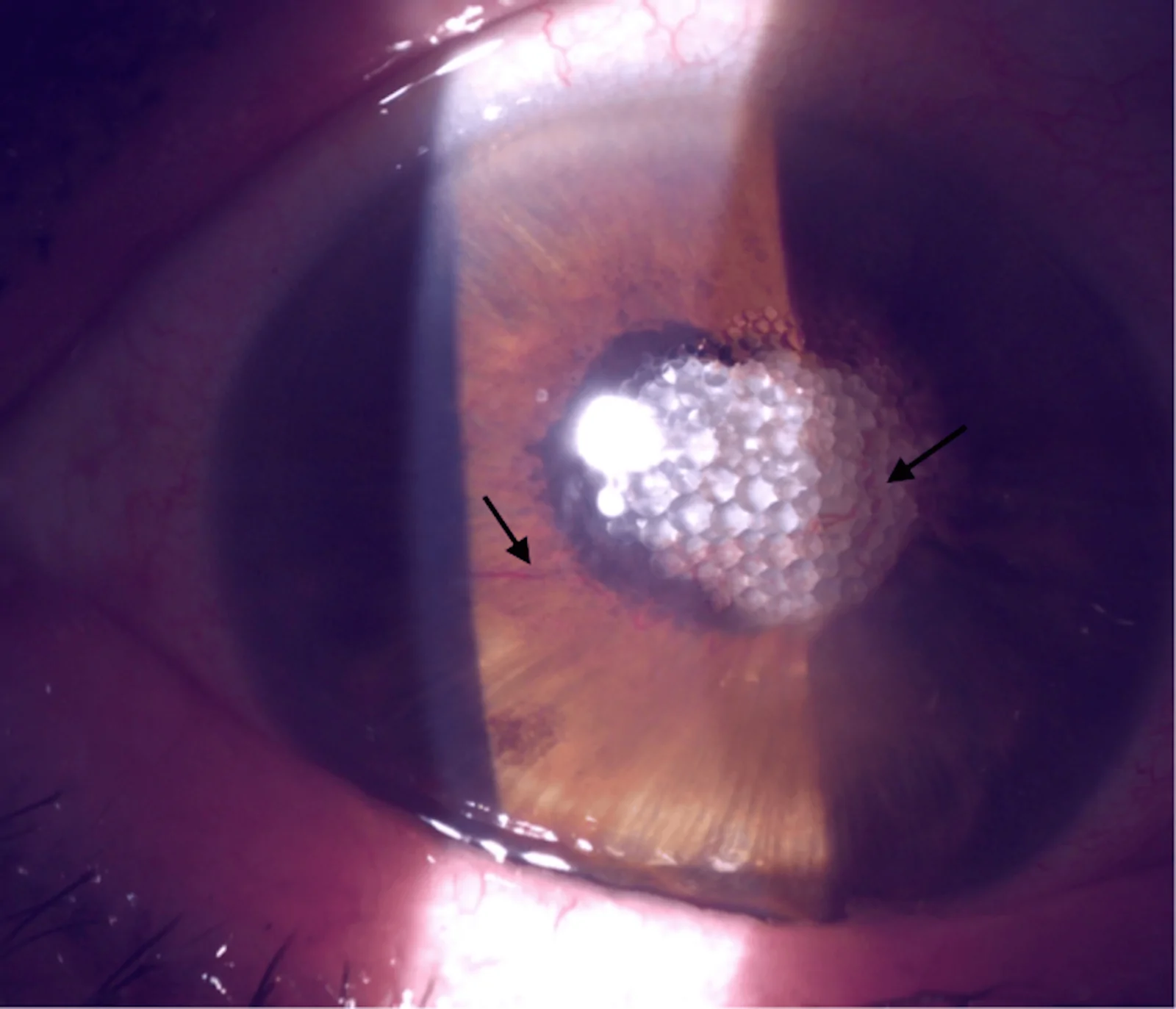
Slit-lamp photograph of silicone oil emulsification with iris rubeosis and intralenticular neovascularization (16x magnification).

The patient elected to continue care with her established retinal specialist. No subsequent follow-up with our service was available, and therefore, the clinical course and outcome of the right eye could not be determined. The patient was advised regarding the importance of continued retinal follow-up and monitoring of the right eye for pain, elevated intraocular pressure/neovascular glaucoma, and progression toward phthisis bulbi.

## Discussion

In 1962, Cibis and colleagues introduced silicone oil as an intraocular tamponade for complicated retinal detachment repair [7]. Silicone oil provides retinal tamponade through interfacial surface tension at the oil-aqueous interface, which resists fluid passage through the retinal break, while buoyancy helps maintain contact between the oil and retina. The goal of intraocular tamponade is to prevent a relapse of retinal detachment. Examples of intraocular tamponade agents include conventional and heavy (denser-than-water) silicone oils, as well as perfluorocarbon liquids [8]. Although generally well tolerated, anterior migration and emulsification of the silicone oil may occur, particularly in pseudophakic eyes with compromised posterior capsular or zonular integrity. In the present case, a compromised posterior capsule permitted silicone oil to migrate into the anterior chamber, where emulsification was observed [9].

Although silicone oil is relatively safe in the eye indefinitely and chemically inactive, it is intended to be used temporarily, as clinical complications are more likely to occur the longer it stays in the eye. Complications include cataract formation, intraocular pressure elevation, corneal edema, and band keratopathy, with postoperative elevation of intraocular pressure being the most reported [10]. Reported rates of postoperative IOP elevation vary widely, ranging from approximately 2.2% to 56% in early studies [11]. This range should be interpreted cautiously because the studies used different definitions of IOP elevation, including absolute IOP thresholds and increases relative to baseline, assessed outcomes at different postoperative intervals, and included heterogeneous patient populations with varying lens status and surgical characteristics. More recent studies using standardized definitions and contemporary surgical techniques have generally reported lower rates of postoperative IOP elevation. This elevation is due to a variety of confounding factors, including the viscoelastic properties of silicone oil and high intraocular pressure.

Historically, silicone oil removal has often been considered within approximately 3-6 months once the retina is stable; however, the optimal timing remains individualized and depends on retinal stability, the severity of proliferative vitreoretinopathy, the risk of recurrent retinal detachment, intraocular pressure, and other oil-related complications. In selected eyes with severe or recurrent pathology, longer-term tamponade may be appropriate. Thus, silicone oil removal should be considered once the retina is sufficiently stable and the anticipated benefits of continued tamponade no longer outweigh the risks of prolonged oil retention [3,8,12]. Routine intraocular pressure monitoring post-surgery is necessary to carefully monitor patients and mitigate potential complications. In one study of complex retinal detachment surgery, tamponade duration was not associated with final anatomic outcomes or retinal redetachment [13]. This finding should be distinguished from evidence linking prolonged tamponade and elevated IOP with unexplained visual loss [12].

The risk of developing complications is higher when injecting silicone oil into an aphakic or pseudophakic eye and is correlated with the integrity of the posterior capsule and lens zonules [8]. When silicone oil is injected into an aphakic or pseudophakic eye with a ruptured posterior capsule, the oil has direct access to the anterior chamber, and anterior segment complications can result. If the zonules have been damaged or weakened due to ocular trauma, high myopia, or the result of multiple surgeries, silicone oil will have easier access into the anterior chamber. Once the oil is in the anterior chamber, it may disperse or emulsify; dispersion comprises larger oil globules, and emulsification results in finer bubble formation [14]. Another potential complication is the anterior displacement of the iris diaphragm, which can lead to increased intraocular pressure, corneal decompensation, pain, and eventual angle-closure glaucoma. This can occur from a surplus of silicone oil (silicone overfill) that may result in the complete filling of the anterior chamber [11]. Most complications associated with silicone oil are a result of emulsification [14].

The patient presented here had a complex ocular history resulting in a sequela of complications. She had a retinal detachment that resulted in postoperative proliferative vitreoretinopathy. The surgical repair involved a pars plana vitrectomy with silicone oil. Retinal detachment repair may result in complications, including proliferative vitreoretinopathy, resulting in the growth of fibrotic membranes in the vitreous that contract and expand onto the retinal borders, resulting in fibrosis [15]. Proliferative vitreoretinopathy is believed to be a form of abnormal wound healing involving retinal pigment epithelium and retinal and inflammatory cells [15]. Additionally, the patient was pseudophakic with a ruptured posterior capsule from a complicated cataract extraction. Thus, after silicone oil was placed in her eye, the oil migrated into the anterior chamber with resultant silicone oil emulsification. After retinal detachment repair, the patient developed rubeosis iridis and intralenticular neovascularization.

Neovascularization can affect any part of the eye, including the retina, iris, anterior chamber angle, and cornea; however, due to the avascular nature of the lens, the lens is rarely affected. A compromised lens capsule (secondary to trauma, surgery, pseudophakia, or hypermature cataract) may increase the propensity for developing intralenticular neovascularization. Anterior segment neovascularization may develop in eyes with chronic posterior segment ischemia from conditions such as chronic peripheral retinal detachment or proliferative vitreoretinopathy. Ischemia stimulates the release of VEGF and erythropoietin. Diffusible VEGF can follow a concentration gradient from the vitreous cavity toward the anterior segment, where it may contribute to anterior segment neovascularization [3]. Erythropoietin is also an ischemia-induced angiogenic factor and has been shown to be elevated in the vitreous in ischemic retinal disease. To prevent neovascularization, angiogenic and antiangiogenic factors within the eye must remain balanced. Because the lens is an avascular structure, intralenticular neovascularization is rare. Intralenticular neovascularization is a rare manifestation of severe ocular ischemia and has previously been reported in association with chronic retinal detachment and proliferative diabetic retinopathy [4]. In the present case, anterior segment neovascularization occurred in the setting of chronic retinal detachment and proliferative vitreoretinopathy, with concurrent silicone oil migration into the anterior segment.

Patel et al. reported a case of intralenticular neovascularization in a 61-year-old diabetic male with a past diagnosis of proliferative diabetic retinopathy and bilateral tractional retinal detachment that had only been repaired in his left eye. His right eye had a hypermature cataract with iris and lenticular neovascularization. It was surmised that the proliferative diabetic retinopathy and severe ocular ischemia from chronic retinal detachment resulted in intralenticular neovascularization. The patient had no light perception in the right eye and ultimately lost all vision [4]. A case series of intralenticular neovascularization subsequent to traumatic cataract formation has been reported as well. The four reported patients developed hypermature cataracts and phacolytic inflammation following ocular trauma. Subsequent neovascularization of the lens developed [5]. These patients were referred for cataract surgery with variable final outcomes. An additional case of intralenticular neovascularization was cited in a young Asian female who presented with a coloboma, traumatic cataract, and retinal detachment. The proposed mechanism similarly involved chronic ocular inflammation secondary to injury and resultant ocular inflammation [6].

Proliferative vitreoretinopathy and a high encircling scleral buckle may exacerbate anterior segment ischemia, further predisposing an eye to anterior segment neovascularization. In cases of surgical repair for proliferative vitreoretinopathy, complications such as iris rubeosis can occur. It is often associated with a peripheral retinal detachment or residual anterior retina after retinectomy [3,16]. Iris rubeosis is commonly seen in conditions including diabetic retinopathy and vein occlusion; however, hypoxia of the detached retina is the stimulant for neovascularization of the anterior segment and subsequent rubeosis. VEGF is released in the presence of an ischemic retina, resulting in neovascularization. VEGF can migrate anteriorly into the aqueous, leading to anterior segment neovascularization. This stimulates the growth of new vessels at the pupillary margin and throughout the anterior portion of the eye.

The differential diagnosis for anterior segment neovascularization in this patient includes proliferative diabetic retinopathy, retinal vein occlusion, ocular ischemic syndrome, chronic retinal detachment with proliferative vitreoretinopathy, and severe intraocular inflammation or trauma. Proliferative diabetic retinopathy was less likely because the patient had no history of diabetes. Retinal vein occlusion and ocular ischemic syndrome were not documented in the affected eye. In contrast, the patient had a history of chronic retinal detachment with grade C proliferative vitreoretinopathy, providing a plausible source of persistent retinal ischemia and VEGF-mediated anterior segment neovascularization. The compromised posterior capsule and anterior migration of emulsified silicone oil may have further facilitated the observed anterior segment findings. Thus, although the precise contribution of each mechanism cannot be established given the absence of a posterior-segment view, the clinical history supports chronic retinal ischemia associated with retinal detachment/PVR as the most likely underlying driver.

Management of anterior segment neovascularization generally focuses on controlling intraocular pressure and addressing the underlying retinal ischemia and associated VEGF-mediated neovascularization. In the present case, however, the patient had profound vision loss in the affected eye, chronic retinal detachment with proliferative vitreoretinopathy, and a complex history of silicone oil tamponade with anterior migration. She was already under the care of a retinal specialist; therefore, further intervention was deferred, and continued specialist follow-up was recommended. In phacolysis, a surgical lensectomy can be performed to control intraocular pressure. Panretinal laser photocoagulation is effective in combating chronic ocular ischemia. Anti-VEGF agents, including Avastin (Bevacizumab), Lucentis (Ranibizumab), Eylea (Aflibercept), Vabysmo (Faricimab), and Beovu (Brolucizumab), have been proven efficacious in modulating circulating VEGF.

The patient’s report of periocular pain when she previously attempted to discontinue Cosopt also warrants consideration. Although the patient associated discontinuation of the medication with recurrence of pain, this does not establish that the pain was caused by elevated intraocular pressure or that Cosopt was responsible for maintaining the markedly low IOP measured at presentation. In a blind eye with chronic retinal detachment, proliferative vitreoretinopathy, profound hypotony, silicone oil in the anterior chamber, and iris rubeosis, pain may be multifactorial. Potential causes include chronic intraocular inflammation, ciliary body dysfunction, cyclitic membrane formation, corneal decompensation or band keratopathy, and neovascular glaucoma that may have occurred earlier in the disease course. Thus, the history of pain following discontinuation of Cosopt may suggest that the eye had previously experienced pressure-related or inflammatory pain, but the underlying cause cannot be determined from the available information. Management of a blind, painful eye should focus on identifying and treating reversible causes of pain while addressing the underlying ischemic and neovascular processes. If pain persists despite conservative treatment, cyclodestructive procedures or, in refractory cases, enucleation or evisceration may be considered.

This case has several limitations. The patient declined additional posterior segment imaging, limiting our ability to evaluate the chronic retinal detachment, choroid, and ciliary body and to further characterize the cause of the profound hypotony. Although attempts were made to obtain the most recent outside records from the retina specialist, they were unavailable. Ultrasound biomicroscopy and anterior segment OCT were considered but were not performed, limiting further characterization of the precise anatomical location and extent of the anterior segment and lenticular neovascularization. Consequently, the diagnosis and proposed mechanisms are based primarily on clinical examination and slit-lamp photography.

## Conclusions

Silicone oil remains an important tamponade agent in the management of complex retinal detachments, but anterior migration and emulsification can result in anterior segment complications. We present an unusual constellation of emulsified silicone oil in the anterior chamber, iris rubeosis, and neovascularization involving the lens/capsular region in a pseudophakic eye with a history of retinal detachment complicated by grade C proliferative vitreoretinopathy and silicone oil tamponade. Chronic retinal ischemia associated with retinal detachment and PVR provides a plausible explanation for the anterior segment neovascularization; however, the precise causal contribution of retinal ischemia, PVR, prior surgery, and silicone oil migration cannot be established from the available data. This case highlights the importance of recognizing anterior segment neovascularization and silicone oil-related changes in eyes with complex retinal detachment histories.
